# Evaluation of Bipolar Disorder in Several Relapses over Time Using Generalized Estimating Equations

**Published:** 2017-07

**Authors:** Payam Amini, Roya Najafi-Vosough, Ali Ghaleiha, Hossein Mahjub

**Affiliations:** 1 Department of Epidemiology and Reproductive Health, Reproductive Epidemiology Research Center, Royan Institute for Reproductive Biomedicine, ACECR, Tehran, Iran.; 2 Department of Biostatistics & Epidemiology, School of Public Health, Hamadan University of Medical Sciences, Hamadan, Iran.; 3 Behavioral Disorders and Substance Abuse Research Center, Hamadan University of Medical Sciences, Hamadan, Iran.; 4 Department of Biostatistics & Epidemiology, School of Public Health and Research Center for Health Sciences, Hamadan University of Medical Sciences, Hamadan, Iran.

**Keywords:** *Bipolar Disorder*, *Generalized Estimating Equation Model*, *Iran*

## Abstract

**Objective:** Bipolar disorder is defined as a common and severe chronic disorder that causes several problems in a person's psychosocial functioning. This study aimed at modeling the development of bipolar disorder episodes using its determinant risk factors over time.

**Method:** This retrospective cohort study was conducted in Hamadan province, the West of Iran, from April 2008 to September 2014. In this study, 124 patients with bipolar disorder (both Type I and Type II) participated. All patients had experienced 4 relapses. Generalized Estimating Equation (GEE) was used to model bipolar disorder episodes, and significance level was set at 0.05.

**Results:** The mean (±SD) age of the patients was 33.2 (±11.55). Males were more likely to experience mania than depression compared to females (odds ratio = 2.30, 95% CI (1.37-3.86)). Patients who received psychotherapy plus medicine were less likely to experience mania than depression compared to drug receivers (odds ratio = 0.39, 95% CI (0.18-0.88)). In the spring, patients were more likely to experience mania than depression compared to the winter (OR = 2.22, 95% CI (1.18-4.19)).

**Conclusion:** The results of the present study revealed that among bipolar disorder patients in the West of Iran, mania was much more prevalent than depression and mixed episodes. Moreover, it was found that sex, treatment, and season can determine the episodes of bipolar disorder.

Bipolar disorder is defined as a severe, chronic, and disabling mental disorder with significant ‎effects on the patient, economy, and the society (1, 2). According to the fourth edition of ‎Diagnostic and Statistical Manual of Mental Disorders Text Revision (DSM-IV-TR), two ‎major categories of bipolar disorder exist: Bipolar I disorder and bipolar II disorder (3). This brain disorder causes many problems some of which are as follow: disability to perform daily activities, diminished daily functioning, being easily distracted, experiencing changes in appetite, unstable emotional moods and risky behaviors, extreme changes in sleep patterns and energy, and relationship difficulties. This mental health problem leads to costs for the patients, their family, and the society (4).

Bipolar disorder is a worldwide concern. Lifetime prevalence rates of bipolar Type I and bipolar spectrum disorders are estimated to range from 0.2% to 1.6% and 3% to 7%, respectively (5). Over 90% of the patients experience recurrences of bipolar disorders in the same or different episode in their lifetime (6).

Almost 60% and 75% of patients experience relapse within 2 and 5 years, respectively (7, 8). Based on World Health Organization reports, bipolar disorder is the sixth leading cause of life-long disability worldwide (9). 

Bipolar disorder is affected by several risk factors such as genetics and medications (10). 

An untreated bipolar disorder patient is at a high risk of suicide and many other adverse consequences (11).

However, detecting the milder forms of bipolar disorder is not straightforward and this leads to underestimation the incidence rate of bipolar disorder. Although there is lack of information about the true incidence rate of bipolar disorder, determining and controlling the risk factors for the 3 episodes of bipolar disorders can considerably decrease the potential adverse outcomes (12-14).

In this study, we aimed at modeling the development of 3 episodes of bipolar disorders (mania, depression, and mixed episodes) using 4 relapses for each patient over time. Several risk factors were used to model and predict the relapse of bipolar disorder episodes, and to do so, generalized estimating equations method was applied.

## Materials and Methods

***Data Collection***

This retrospective cohort study was conducted in Hamadan province, the West of Iran, from April 2008 to September 2014. Patients who were hospitalized due to bipolar disorder (both Type I and Type II) relapses were recruited in this study. The patients were sampled randomly from Farshchian hospital in Hamadan. This hospital is the only psychiatric hospital in Hamadan province. The data were extracted from patients' records. A checklist of items was prepared according to the context of the patients' records and clinical examination of the psychiatrists. The checklist included data on sex (male/ female), job (employed/ unemployed), family history of mood disorder (yes/ no), and type of treatment (drug/ drug and ECT/ drug and psychotherapy), and season (spring/ summer/ autumn/ winter). Moreover, the episodes of bipolar disorder were the response variable (a nominal variable with 3 levels of depression, mania and mixed). More details about the data have been well- explained in Najafi-Vosough et al. (15).

***Statistical Analysis***

The data were analyzed using both adjusted and unadjusted approaches. In the unadjusted methods, Chi-square test was used to investigate any association between bipolar disorder episodes and the risk factors. 

Because the data were collected longitudinally, the unadjusted approaches were not able to consider the variation caused by the repeated measurements over time for the same subject. The adjusted approach was used to consider the interaction among the predictors. Moreover, the association between the repeated episodes for the same subject was taken into account using this approach. Longitudinal modeling approach was applied due to longitudinal recording of bipolar disorder episodes. Among different approaches, the GEE method is frequently recruited according to its ability to model any type of variables (16, 17). This modelling approach provides the possibility of predicting the bipolar disorders episodes. This population average method is an extension of Generalized Linear Models (GLM), which deals with any kind of response variable (e.g. continuous, count, binary). This method models covariance pattern of repeated measurements and the mean response in 2 different steps. Interpretation of the results in the GEE is straightforward. The GEE approach presents a statistical formula (model) in which the mean response can be predicted using the covariates values and their estimated coefficients (18, 19). Because the response variable was nominal, a logistic regression model using the GEE was applied. In the following formula, c is mania, mixed episodes and the reference category is assumed as depression, γ is threshold, X is the matrix of covariates and factors, and β is the vector of coefficients. 


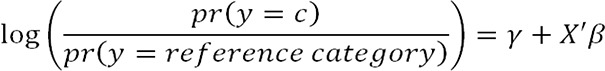


In this study, the GEE was applied to evaluate the impact of sex, job, smoking, treatment, season, family history of mood disorder, and time (4 relapses) on the stage of bipolar disorder. The interpretations were based on odds ratio (OR) so that e^β was odds of a risk factor in mania, or mixed on depression (20). The results are presented as the estimate of the covariate/factor coefficients, their standard error, and 95% confidence interval for the odds ratio. A confidence interval including 1 is in accordance with a p-value more than 0.05. Statistical analysis was performed using SPSS Version 16.0 (Chicago, IL) and package lme4 in R.3.2.3 software (An open source programming environment for data analysis). A significance level of 0.05 was considered for the tests.

## Results

A total of 124 patients were followed in 4 relapses of bipolar disorders. The mean (± standard deviation) age of the patients was 33.2 (±11.55). Table 1 demonstrates the details about the frequency and percentage of categorical variables. The results in Table 2 display no significant differences among the proportions of different episodes in several relapses.

Table 3 demonstrates the GEE results. The table contains 7 columns including independent variable and its categories, estimated coefficient comparing mania to depression in different categories of the independent variable, estimated standard error, the odds ratio (95% CI), estimated coefficient comparing mixed episodes to depression in different categories of the independent variable, estimated standard error, and the odds ratio (95% CI), respectively. According to the 95% confidence interval for the odds ratios, sex, family history of mood disorder, treatment, and season were significant predictive variables for the episodes of bipolar disorders.

According to Table 3, the odds of mania to depression episodes was 2.30 (95% CI: 1.37-3.85) times higher among the males compared to the females. In other words, males were more likely to experience mania (given depression as the reference category) compared to females. 

No significant association was found between bipolar disorder episodes and job. However, being employed decreased the odds of depression compared to both mania and mixed episodes. Mania, in comparison to depression, was more common among employed patients. Based on Table 3, among the employed cases, the odds of mania to depression was 1.53 times higher than the unemployed cases. However, the relationship was not statistically significant. 

Those patients who only took medication experienced depression (compared to mania) episode of bipolar disorder more than those who received both psychotherapy and medication (OR = 0.39, 95% CI: 0.17-0.88). Although the odds of mixed episode (compared to depression episode) in psychotherapy plus medicine group was 1.05 (95% CI: 0.33-3.35) than the only medicine group, this association was not statistically sensible. The patients who received medicines and ECT were more prone to experience mania than depression with the odds ratio of 1.07 (95% CI: 0.64-1.76). Moreover, patients with both medication and ECT were less likely to experience mixed episodes than depression, with the odds ratio of 0.97 (95% CI: 0.41-2.30).

 Smoking was not associated with bipolar disorder episodes. However, mania (given depression as a reference category) was less common among smokers compared to nonsmokers (OR = 0.66, 95% CI: 0.34-1.11). In addition, the smokers were more prone to be in the mixed episode than depression, with the odds ratio of 1.17. 

The spring, compared to the winter, was more risky for experiencing mania than depression. The odds ratio of mania to depression in the spring was 2.22 times than in the winter. No statistical change of the episodes was observed over time for the patients.

**Table1:** The frequency distribution of bipolar disorder patients’ characteristics

**Variable**	**Category**	**Frequency**	**Percent**
Sex	male	93	75.0
	female	31	25.0
Job	Employed	60	49.2
	Unemployed	64	50.8
Family history of mood disorder†	Yes	42	33.9
	No	82	66.1
Smoking	Yes	27	21.8
	No	97	78.2
Treatment	Drug	77	62
	ECT and Drug	37	29.8
	Psychotherapy and Drug	10	8.2
Season	Spring	34	27.4
	Summer	35	28.2
	Autumn	23	18.6
	Winter	32	25.8

† Family History with Mood Disorder Included Bipolar or Unipolar Disorder

**Table2:** Frequency (Percent) of Bipolar Disorders Episodes in Different Relapses

**Relapse**	**Bipolar Disorder**			**P-value based on ** **ϰ** ^2^
	**Manic**	**Depression**	**Mixed**	
first	73(67.6)	29(26.9)	6(5.5)	0.508
Second	72(62.1)	32(27.6)	12(10.3)	
Thirds	65(56)	41(35.3)	10(8.7)	
Fourth	74(65.5)	29(25.7)	10(8.8)	

**Table3:** The Results of GEE† Logistic Regression Evaluation of Bipolar Disorder

**Variable**	**Mania to Depression**			**Mixed to Depression**		
	**B** ‡	**SE** §	**OR** ¶ **(95% CI**¶¶**)**	**B** ‡	**SE** §	**OR** ¶ **(95% CI**¶¶**)**
**Sex**						
Male	0.833	0.263	2.30 (1.37-3.85)	0.252	0.429	1.28(0.55-2.99)
Female	reference category					
**Job**						
Employed	0.427	0.232	1.53 (0.97-2.42)	0.312	0.391	1.36 (0.62-2.95)
Unemployed	reference category					
**Family history of mood disorder**						
Yes	0.776	0.252	2.16 (1.31-3.54)	0.601	0.407	1.82(0.81-4.05)
No	reference category					
**Smoking**						
Yes	-0.474	0.297	0.66(0.34-1.11)	0.158	0.462	1.17(0.47-2.90)
No	reference category					
**Treatment**						
Psychotherapy + Drug	-0.923	0.405	0.39(0.17-0.88)	0.052	0.589	1.05 (0.33-3.35)
ECT + Drug	0.068	0.255	1.07(0.64-1.76)	-0.021	0.435	0.97(0.41-2.30)
Drug	reference category					
**Season**						
Autumn	0.207	0.307	1.23 (0.67-2.25)	0.266	0.513	1.30(0.47-3.57)
spring	0.797	0.323	2.22 (1.17-4.19)	0.113	0.573	1.12(0.36-3.45)
Summer	0.325	0.307	1.38 (0.75-2.53)	0.198	0.516	1.22(0.44-3.36)
winter	reference category					
time	-0.005	0.005	0.99 (0.98-1.01)	-0.016	0.010	0.98 (0.96-1.01)

† Generalized Estimating Equations,

‡ Estimated coefficients,

§ Standard Error,

¶ Odds Ratio,

¶¶ Confidence Interval

## Discussion

This study examined the impact of potential risk factors on the development of bipolar disorder episodes over four relapses. Our findings revealed that the rate of mania was more than other 2 episodes. The higher proportion of manic patients in this study might be due to the difficulty of providing care for the patients in their families. Therefore, they were hospitalized more and earlier than depressed patients. In the GEE analysis method, depression was assumed as the reference category and was compared to the other 2 episodes. Many studies found depression as the predominant episode of bipolar disorders (21, 22). Besides, some authors found mania to be more common compared to other episodes (23). In 2005, Post and Robert reported that most of the patients remain more depressed, and this leads to a higher risk of suicide, impairment in work, society, and family life (24).Our study revealed that the odds of mania to depression were almost twice among males than in females. In our study, hospitalization was more common among manic male patients due to more declines in functioning. In a study conducted by Suppes et al. in 2005, the prevalence of mixed symptoms was assessed. After conducting a naturalistic prospective study, they found that mixed hypomania is more common in females (25). Other studies demonstrated a great risk of bipolar disorders in females than in males (26). Kupka et al. found that the risk of bipolar disorder in females was significantly higher than in males (27). Similar to the results of our study, Nivoli et al. assessed sex differences in a cohort study of 604 bipolar patients in 2010. He showed that, in contrast to males, females experience depression more than mania (28).

In our study, some patients received psychotherapy in addition to routine medications. The results of our study revealed a significant higher rate of mania to depression episodes for those who received medications compared to those who received both psychotherapy and medicine. Swartz et al., in reviewing the evidences for psychotherapy in adults with bipolar disorder, demonstrated that bipolar disorder-specific psychotherapies, in addition to medication, show advantages over medication alone (29, 30). Cakir and Ozerdem evaluated the psychotherapeutic and psychosocial approaches in bipolar disorder using a systematic review. They realized that psychosocial and psychotherapeutic interventions combined with drug treatment seem to be effective in preventing relapses (31). 

 This study found that bipolar disorder relapses were more common in the spring. Spring and winter were the only 2 seasons with significant differences in prevalence rates of the episodes. Thus, spring was introduced as a risk factor for bipolar disorder episodes. Patients in our study experienced mania and mixed episodes in the spring approximately twice than in the winter. Several studies investigated the seasonal and environmental effects on bipolar disorder. Hakkarainen et al. studied the seasonal changes among twins with bipolar disorder. They found that mania and depression were more popular in autumn and winter, respectively (32). In a systematic review by Geoffroy et al. in 2014, the association of bipolar disorders and seasonality was assessed. Their study revealed a higher prevalence of mania in the spring and summer, while depression was commonly observed in the winter (33). Akhter et al. checked seasonal variation of manic and depressive symptoms in bipolar disorder in 2013. Using a retrospective study including 314 patients, they found that winter and autumn were risky seasons for bipolar disorder patients (34).

Longitudinal methods are widely used in medical areas. Repeated measurements over time cause a type of variation in which a subject specific effect must be considered. Ignoring the mentioned variation increases the residual errors in models and results in invalid estimations. Two sources of variation are present in longitudinal data. The first source is the difference among cases in the study, and the second is the variation caused by the repeated measurements over time. All the statistical methods cope with the first source, while the second source of variation can only be dealt by longitudinal approaches. To prevent any bias, longitudinal methods should be applied to consider the variation caused by the repeated measures over time. The data of this study were analyzed using GEE method. MacKinnon et al. used GEE for comorbid bipolar disorder and panic disorder in families with a high prevalence of bipolar disorder (35). For sequencing substance use and affective morbidity in 166 first-episode bipolar I disorder patients, Baethge et al. used generalized estimating equations (36). Moreover, many studies have applied GEE to analyze longitudinal responses (37-39).

## Limitations

The limitations of this study were the absence of some data in patients' records, and its retrospective design. Despite these limitations, the current study revealed the development of bipolar disorder over time.

## Conclusion

The results of this study, using GEE method, revealed that mania is much more prevalent than depression or mixed bipolar disorder among patients in the West of Iran. Moreover, it was found that several episodes of bipolar disorder are affected by sex, treatment, and the seasons of relapse.
